# Evaluation and implementation of a mannequin-based surgical simulator for margin-involving eyelid laceration repair – a pilot study

**DOI:** 10.1186/s12909-021-02600-3

**Published:** 2021-03-19

**Authors:** Jiawei Zhao, Meleha Ahmad, Emily W. Gower, Roxana Fu, Fasika A. Woreta, Shannath L. Merbs

**Affiliations:** 1 Wilmer Eye Institute, Johns Hopkins University School of Medicine, Baltimore, MD USA; 2 Gillings School of Global Public Health and Department of Ophthalmology, University of North Carolina, Chapel Hill, NC USA; 3 Department of Ophthalmology, University of Pittsburgh Medical Center, Pittsburgh, PA USA; 4 Department of Ophthalmology and Visual Sciences, University of Maryland School of Medicine, 419 W. Redwood St., Suite 420, Baltimore, MD 21201 USA

**Keywords:** Surgical simulator, Resident education, Surgical training, Eyelid laceration repair, Oculoplastic surgery, Ophthalmology

## Abstract

**Background:**

Repair of margin-involving eyelid lacerations is a challenge for beginning ophthalmology residents, yet no commercially-available simulation models exist for learning this skill. The objective of the study was to modify a mannequin-based surgical simulator originally developed for trachomatous trichiasis surgery training to teach margin-involving eyelid laceration repair and to evaluate its success within a residency wet-lab environment.

**Methods:**

We modified a previously developed mannequin-based training system for trachomatous trichiasis surgery into a simulator for margin-involving eyelid laceration repair. Six ophthalmology residents from a tertiary care academic institution performed at least one simulated margin-involving eyelid laceration repair using the surgical simulator between September 2019 and March 2020. Each session was video recorded. Two oculoplastic surgeons reviewed the videos in a blinded fashion to assess surgical proficiency using a standardized grading system. Participants were surveyed on their comfort level with eyelid laceration repair pre- and post-completion of simulation. They were also queried on their perceived usefulness of the surgical simulator compared to past methods and experiences.

**Results:**

Six residents completed 11 simulation surgeries. For three residents who completed more than one session, a slight increase in their skills assessment score and a decrease in operative time over two to three simulation sessions were found. Self-reported comfort level with margin-involving eyelid laceration repairs was significantly higher post-simulation compared to pre-simulation (*p* = 0.02). Residents ranked the usefulness of our surgical simulator higher than past methods such as fruit peels, surgical skill boards, gloves, and pig feet (*p* = 0.03) but lower than operating room experience (*p* = 0.02). Residents perceived the surgical simulator to be as useful as cadaver head and emergency department/consult experience.

**Conclusions:**

We developed a surgical simulator for teaching eyelid laceration repair and showed its utility in developing trainees’ surgical skills. Our surgical simulator was rated to be as useful as a cadaver head but is more readily available and cost effective.

**Supplementary Information:**

The online version contains supplementary material available at 10.1186/s12909-021-02600-3.

## Background

Surgical training across multiple specialties, including ophthalmology, has traditionally been based on an apprenticeship model, resulting in significant variability in a trainee’s experience and exposure to different procedures. There has also been a decrease in resident autonomy as rules for ensuring patient safety have increased [1]. A growing number of simulation-based models have become available in ophthalmology allowing for the training and assessment of procedural skills without associated patient risk [2, 3].

Eyelid lacerations associated with ocular and periocular trauma often occur outside of regular clinic hours, when residents take primary call. Therefore, it is important for an ophthalmology resident to be proficient at eyelid laceration repairs early during their training. Eyelid laceration repair has been prioritized as one of the top 10 procedures that should be practiced in a simulation-based manner to achieve proficiency before working with actual patients [4]. Lacerations involving the eyelid margin are particularly challenging to repair, and they require a complex, layered closure with several different suture materials. Familiarity with the steps of the technique and an appreciation for the feel of the tissues involved can help the ophthalmology resident achieve a well-reconstructed eyelid that is both functional and aesthetic.

Very few simulation models exist for teaching oculoplastics procedures. Cadaver-based models are advantageous because of their fidelity and realism, but they have limited applications due to low availability and high cost [5, 6]. A surgical skill board has been employed to teach simple wound closure in a 1-day surgical course run by Moorfields Eye Hospital [7]. Animal models, such as pig eyelid [8] and split pig-head models [9], have been used to teach eyelid laceration repair. However, there can be inconsistency in the quality of the animal tissue and important anatomic differences when compared to the human eyelid.

The aim of this study was to develop and evaluate a cost-effective and reproducible surgical simulator to teach margin-involving eyelid laceration repair. In addition, we investigated the implementation of the simulator within a wet lab training environment.

## Methods

All study procedures were approved by the Institutional Review Board of the Johns Hopkins University School of Medicine and adhered to the requirements of the Health Insurance Portability and Accountability Act.

### Mannequin-based simulator

**Fig. 1:**
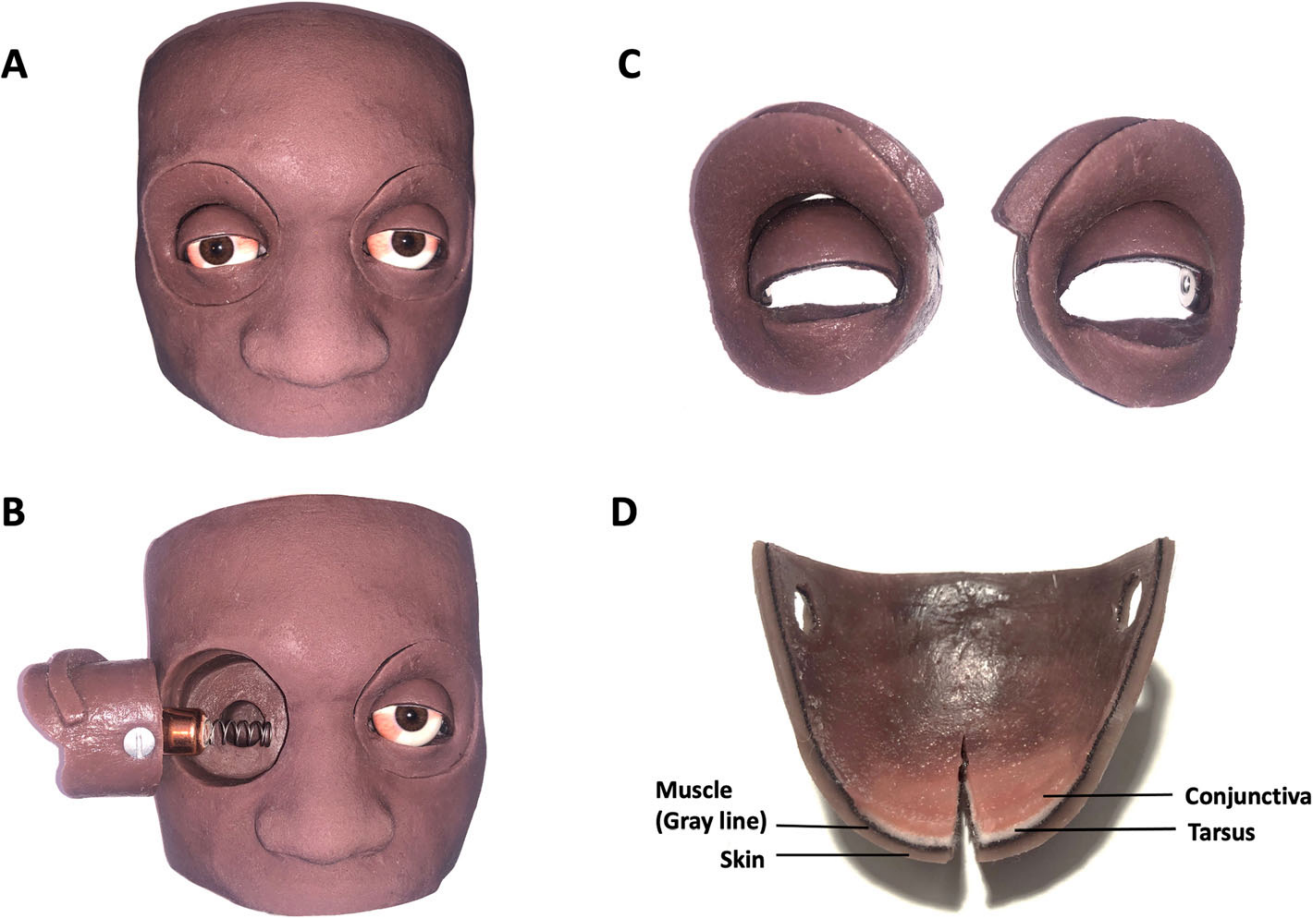
**a** The mannequin-based surgical simulator for margin-involving eyelid laceration repair. **b** The orbit is removable. **c** The eyelid cartridge attaches to the orbit. **d** A vertical incision made in the eyelid demonstrates the four layers of the simulator eyelid that mimic the primary layers of the human eyelid: skin, orbicularis muscle, tarsus and conjunctiva

A mannequin-based training system called the Human Eyelid Analog Device for Surgical Training and skills Reinforcement in Trichiasis (HEAD START, Ho’s Art LLC, Yadkinville, NC) was previously developed for trachomatous trichiasis surgery training [10]. The mannequin head is made out of silicone (Fig. 1a) and has a removable orbit upon which a disposable eyelid cartridge is mounted (Fig. 1b-c). The four layers of the eyelid cartridge mimic the primary layers of the eyelid: skin, muscle (gray line), tarsus and conjunctiva (Fig. 1d). The eyelid can be incised and sutures can be placed, allowing a trainee to perform each step of the surgical procedure as they would in patient with the same type of sutures.

Modifications to the standard HEAD START eyelid cartridge were required to replicate a margin-involving eyelid laceration. Instead of a tapered eyelid margin that simulates cicatricial entropion in the standard trachomatous trichiasis cartridge, a square-edged design replicating the architecture of a normal upper eyelid margin was created. In the new cartridge design, the “gray line,” an important anatomic landmark for laceration repair, is visible. The original model had a tarsal layer that was too friable to hold the partial-thickness tarsal sutures that are used to repair a full-thickness eyelid laceration. Various materials were tried in eyelid cartridge prototypes to attain the greatest similarity to the feel of a human tarsus and to provide the tensile strength required to retain partial-thickness sutures. The final design incorporated fleece fabric as the tarsal layer. The remaining layers were made out of different colors and thicknesses of silicone to represent the skin and conjunctiva, and a fabric layer for the orbicularis (Fig. 1d).

### Perceived need for better surgical simulation model

In September 2019, ophthalmology residents of all postgraduate year (PGY) levels at the Johns Hopkins Hospital were given a questionnaire to assess their level of experience and comfort with repairing simple and margin-involving eyelid lacerations (Supplementary file 1A). Informed consent was obtained from all participants. Comfort with repair was rated on a 5-point Likert scale with 5 being most comfortable, and 1 being least comfortable. Demographic information, including age, gender, PGY level and completion status of the oculoplastics rotation were recorded. Finally, participants were queried on prior methods used to practice eyelid laceration repair and their satisfaction with existing methods. Satisfaction with various practice methods was rated on a 5-point Likert scale with 5 being very satisfied and 1 being very dissatisfied.

### Simulator implementation

Before the first simulation session, participants were given a 1-h didactic session on eyelid anatomy and a demonstration of margin-involving eyelid laceration repair using the surgical simulation model. Documentation outlining the details of each procedural step and use of the simulator was made readily available to participants in the wet lab. Participants were instructed to perform up to 5 simulated margin-involving eyelid laceration repairs using the model, 2 weeks apart. Each session was video recorded. Participants were provided with all the necessary surgical instruments, sutures, phone for video recording, adjustable phone holder, and simulator eyelids. Participants were instructed to wear gloves, to remove any possible identifiers throughout the duration of the recording, and to mute the sound of the recording. Identical, full-thickness vertical incisions of the eyelids were made before they were provided to the participants. Two attending oculoplastic surgeons (S.M. and R.F.) reviewed the laceration repair session videos in a masked fashion and random order. They assessed the surgical proficiency of each participant using a standardized grading system (Supplementary file 2A). Operating time was calculated as the period from loading the first suture to the trimming the ends of the final suture.

### Perceived comfort level post simulation and feedback on simulator

At the end of the pilot study (March 2020), a questionnaire was given to the participants that had completed one or more simulation sessions asking them to rate their comfort level with repair of simple and margin-involving eyelid lacerations post-training with the surgical simulator and the usefulness of different models and experiences for learning margin-involving eyelid laceration repair (Supplementary file 3A). These models and experiences included cadaver head, our surgical simulator, past methods (fruit peels, surgical skill boards, gloves, pig feet), as well as operating room and emergency department/consults experiences. In addition, participants were queried on effectiveness and value of our surgical simulator, and whether they would recommend inclusion of the simulator into a formal oculoplastics curriculum.

### Analysis

The Wilcoxon rank-sum test was used to compare Likert scores of comfort level with eyelid laceration repair pre-simulation and post-simulation. It was also used to compare usefulness of different methods for practicing margin-involving eyelid lacerations. Inter-rater reliability of the surgical skills assessment score of each simulated session was determined using the intraclass correlation coefficient (intraclass correlation coefficient, two-way mixed-effects models, consistency). The Spearman’s rank correlation coefficient was employed to measure correlation between the average surgical skills assessment score and operating time. All statistical analysis was performed using SPSS version 25 (IBM Corp., Armonk, N.Y.). A *p* ≤ 0.05 was considered statistically significant.

## Results

### Perceived need for better surgical simulation model

A total of 10 residents completed the pre-training survey. Their demographic information and level of experience with performing simple, margin-involving, and canalicular-involving eyelid laceration repairs are summarized in Table 1. As expected, before the simulation practice, residents reported slightly more comfort with repair of a simple eyelid laceration versus a margin-involving laceration (median Likert scale 3 vs. 2). Prior to the study, 60% of residents had practiced on cadavers, 40% on fruit peels, 40% on gloves, 40% on pig feet and 20% on a surgical skills board during their training as medical students, interns and/or residents. Median Likert scale satisfaction with past methods was 3.

**Table 1:** Demographic data and level of experience with performing eyelid laceration repairs of the 10 residents that completed the pre-training survey

**Demographic Variable**		**N (%)**
**Gender**	Male	5 (50%)
	Female	5 (50%)
**Training level**	PGY2	5 (50%)
	PGY3	4 (40%)
	PGY4	1 (10%)
**Completion of oculoplastics rotation**	Yes	6 (60%)
	No	3 (30%)
	In progress	1 (10%)
**Number of eyelid laceration repair(s) performed**		**Average (SD)**
**Simple**	PGY 2	0.4 (0.5)
	PGY3 + PGY4	8 (4)
**Margin-involving**	PGY 2	0.2 (0.4)
	PGY3 + PGY4	2 (1)
**Canalicular-involving**	PGY 2	0.2 (0.4)
	PGY3 + PGY4	3 (2)

*PGY* postgraduate year

### Simulator implementation

Out of the 10 residents who completed the pre-training survey, 6 performed at least one simulation session. All 5 PGY2 residents participated (1 performed 3 simulations, 1 performed 2 simulations, and 3 performed 1 simulation). One PGY4 resident performed 3 simulations. The time interval between simulation sessions varied between residents (average = 35 days, SD = 32 days). A total of 11 videos were assessed by the two attending oculoplastic surgeons. The inter-rater reliability among the raters was high (Intraclass Correlation Coefficient = 0.862, *P* = 0.002).

**Fig. 2:**
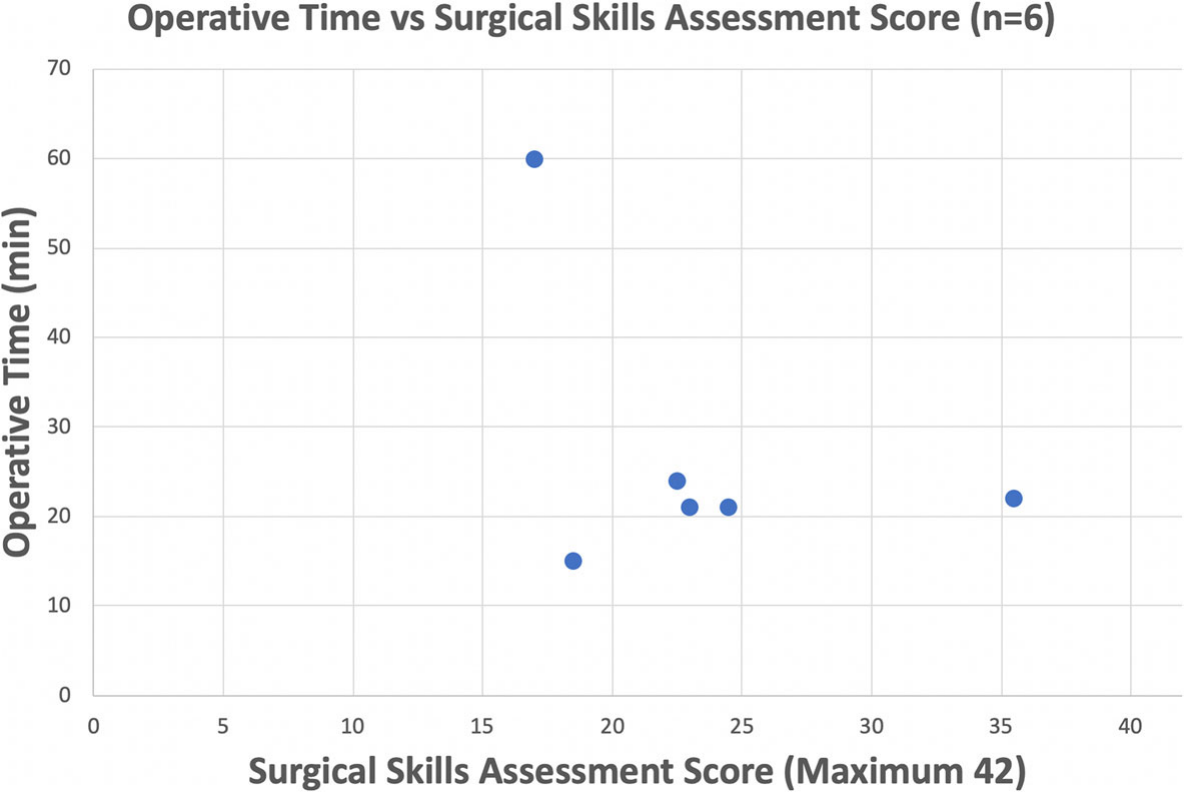
Operative time during first simulation session and average surgical skills assessment score among residents who completed at least one session

**Fig. 3:**
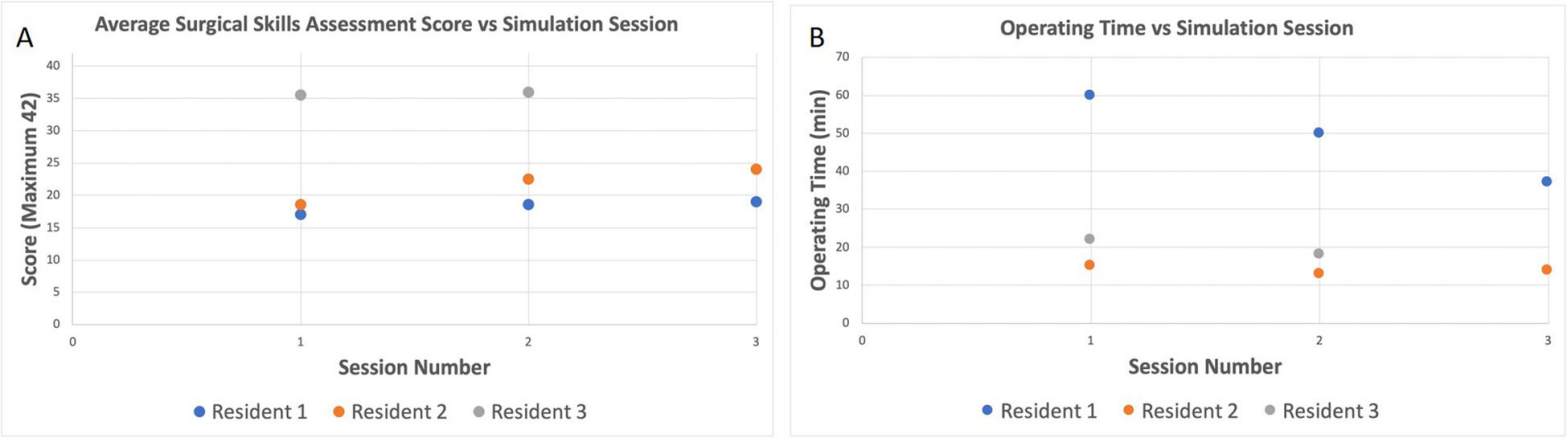
**a** Average session-specific surgical skills assessment score versus simulation session. **b** Operating time versus simulation session

Across all 6 participants, the average surgical skills assessment score for the first session was 24 (SD = 7) out of maximum score of 42. We found no correlation between the first session operating time and the surgical skills assessment score (averaged between 2 attendings) (Spearman’s rank correlation coefficient = − 0.232, *p* = 0.66) (Fig. 2). We plotted each resident’s average surgical skills assessment score versus session number for the 3 residents (Resident 1, a PGY2; Resident 2, a PGY4, and Resident 3, a PGY2) who completed more than one session (Fig. 3a). A slight increase in their skills assessment score across simulation sessions was found while operative time declined across, most notably in Resident 1 (Fig. 3b).

### Perceived comfort level post-training and feedback on simulator

The 6 participants who performed at least 1 simulation session completed the post-training questionnaire. Comfort level with simple eyelid laceration repairs was significantly higher post-simulation compared to pre-simulation (median Likert value 4 vs. 1, *p* = 0.02) and for margin-involving eyelid laceration repair (median Likert value 3 vs 1; p = 0.02). Residents reported that they preferred using our surgical simulator in learning margin-involving laceration repair more than past methods such as fruit peels, surgical skill boards, gloves, and pig feet (*p* = 0.03), and they felt that the simulator was as useful as cadaver heads and emergency department/consult experience (Table 2).

**Table 2:** Comparison of usefulness of the mannequin-based surgical simulator to other methods and experiences in learning margin-involving eyelid laceration repair

	Median Likert Scale^**a**^	***P*** value^**b**^
**Surgical simulator**	2.5	–
**Operating room**	5	0.02
**Emergency department/consult**	4	0.07
**Cadaver head**	2.5	0.74
**Past methods**	1	0.03

^a^Usefulness of each method was ranked on a 5-point Likert scale (1 = least useful, 5 = most useful)

^b^Likert scale of our surgical simulator was compared to operating room, emergency room/consults, cadaver head and past methods (fruit peels, surgical skill boards, gloves, pig feet) using Wilcoxon rank-sum test

All 6 participants were somewhat satisfied or extremely satisfied with using the surgical simulator for suturing practice and for learning margin-involving eyelid laceration repairs (Table 3). Fifty percent of participants recommended including the simulator into the formal oculoplastics curriculum. The remainder of the participants who responded with “Maybe” commented that they would have had a favorable response with curriculum integration if time was specifically allocated for these practice sessions during the oculoplastic surgery rotation. More variation in responses was seen regarding the likelihood of the participants to use this simulator for future eyelid laceration practice.

**Table 3:** Post-simulation questionnaire responses (*n* = 6)

	Satisfaction with effectiveness of simulator for margin-involving laceration repair^**a**^	Satisfaction with effectiveness of simulator for suturing practice^**a**^	Realism of laceration repair with simulator compared to real-life^**a**^	Likelihood of using simulator for future practices^**a**^	Inclusion of simulator into formal curriculum^**b**^
**Likert Scale**	**N (%)**	**N (%)**	**N (%)**	**N (%)**	**N (%)**
1	0 (0)	0 (0)	0 (0)	0 (0)	0 (0)
2	0 (0)	0 (0)	1 (16.7)	1 (16.7)	3 (50)
3	0 (0)	0 (0)	2 (33.3)	3 (50)	3 (50)
4	3 (50)	3 (50)	3 (50)	1 (16.7)	
5	3 (50)	3 (50)	0 (0)	1 (16.7)	

^a^Satisfaction with effectiveness, realism and likelihood of future use of simulator were rated on a 5-point Likert scale (1 = extremely unsatisfied/very unrealistic/extremely unlikely, 5 = extremely satisfied/very realistic/extremely likely)

^b^There were 3 answer choices for the question regarding the inclusion of simulator into formal oculoplastics curriculum (1 = No, 2 = Maybe, 3 = Yes)

## Discussion

A rising demand for increased patient safety has resulted in the rapid growth of simulation-based surgery models in ophthalmology training [2, 3]. Prior studies have demonstrated improvement in operating room performance with the use of surgical simulation [11–13]. This study successfully converted a mannequin-based surgical simulator for trachoma surgery into a simulator for margin-involving eyelid laceration repair and evaluated its usefulness to teach laceration repair. The simulator provided a platform for practicing all the necessary steps of the procedure and permitted evaluation of trainee’s technical performance.

Overall, the feedback regarding the mannequin-based surgical simulator was positive. All participants were satisfied with using it for suturing and for practicing margin-involving eyelid laceration repairs. As a model to teach repair of margin-involving eyelid lacerations, the surgical simulator was perceived to be equivalent to the emergency department/consult experience and cadaver head, but inferior to operating room experiences. It was perceived as superior to past methods used, which included fruit peels, gloves, surgical skill boards, and pig feet. Half of the participants indicated that practice sessions with the surgical simulator should be part of the formal oculoplastics curriculum while the rest were ambivalent. Residents commented in the Post-Training Survey (Supplementary file 3A) that the surgical simulator was most useful for residents at the beginning of their training. In this way, it can help residents gain experience and confidence with use of surgical instruments and different suturing techniques before moving on to real-life experiences, such as the operating room and emergency room.

Animal models have been utilized to teach eyelid laceration repair. Pfaff [8] described the use of pig eyelids. This model involves half of a rubber ball to mimic the convexity of the globe, and steel screws arranged to simulate canthal tendons and the arcus marginalis. Similarly, Kersey [9] reported the use of a split pig-head model, which involves a vertical midline sagittal section of a pig head. On gross and histologic examination, human and pig eyelids were found to be similar. Nevertheless, there are important differences, including thicker skin and additional loose connective tissue in the pig eyelid, and the technical performance of the trainees using their models was not assessed.

Although the usefulness of the mannequin-based surgical simulator and cadaver head were rated to be equivalent in our study, the costs are very different. The reusable mannequin head has a one-time cost of $425, and the eyelid cartridge, the only part of the surgical simulator that needs to be replaced between simulation practices, costs $12.50. This is in contrast to a cadaver, which can cost from $200 to upward of $5000 in processing fees per pair of eyelids [14, 15]. Our surgical simulator is a safer and a more easily manageable alternative to cadaver tissue, and is readily available and can be used in more settings.

Our surgical simulator was successfully integrated into a wet lab environment following a didactic teaching session. Participants’ surgical skills were assessed with a standardized grading system, which showed high inter-rater agreement. Time to complete a procedure is frequently used as an indicator of surgical experience [16, 17]. We did find that two of the three residents with more than one simulation session showed reduction in operative time between sessions. However, when analyzing the first simulation session in all 6 residents, we did not find a correlation between operative time and surgical skills assessment score in our cohort. Similar results were reported in a cleft palate surgery simulator, where operative time did not correlate with level of experience [18]. The researchers postulated that with increasing experience came greater appreciation for difficult surgical steps and the consequence of suboptimal repair [18].

Limitations of this study include the small sample size and a single institution. None of the PGY3 residents performed a simulation session, given they had already completed the oculoplastics rotation and were concentrating on learning cataract surgery. Additionally, they had already performed laceration repairs in the emergency department and operating room, so there was lower motivation to practice on a surgical simulator. The suggested number of simulation sessions was five; however, none of the participating residents was able to complete more than three sessions. This is likely due to the busy clinical demands of the PGY2 year. A possible solution is to build dedicated time for the simulation practices into the resident schedule at the beginning of residency. Many of the mechanical properties of the surgical simulator are remarkably similar to the human eyelid; however, the eyelid obviously does not bleed and the tissue planes do not separate the way they do in a human eyelid. At the time the residents completed the pre-training questionnaire about laceration repair experience, 3 of the 5 PGY-2 residents had not completed the oculoplastics rotation, but at the time of the first simulation session, only 1 of the 5 residents (Resident 1 in Fig. 3) had not completed the oculoplastics rotation. Although anecdotal, it is interesting to note that Resident 1, who had not started the oculoplastics rotation at the time of the first simulation session, had the longest initial simulation session. He/she subsequently reduced their session time by almost 50% by the third session. This highlights the particular usefulness of the model with inexperienced residents.

A multicenter study of this novel mannequin-based training system is ongoing. The training system has been integrated into the orientation curriculum at two different institutions, and nine PGY-2 residents have been invited to participate. This is particularly timely given the importance of increased caution with person-to-person contact during the SARS-CoV2 pandemic and resulting limitations in residents’ direct patient care.

## Conclusion

In this study, we demonstrated the usefulness of a surgical simulator for teaching eyelid laceration repairs and showed its utility for developing trainees’ surgical skills. We successfully used this model to further evaluate trainees’ technical performance with a standardized grading system with high inter-rater reliability. This surgical simulator can be used to augment resident’s training experience and allows for targeted supervision and independent self-reflection before moving onto live surgery.

## Supplementary Information

**Additional file 1: Supplementary file 1A.** Pre-Training Survey

**Additional file 2: Supplementary file 2A.** Surgical skills assessment tool for evaluating proficiency in performing margin-involving eyelid lacerations

**Additional file 3: Supplementary file 3A.** Post-training survey

## Data Availability

All data generated or analyzed during this study are included in this published article.

## Abbreviations

HEAD START: Human Eyelid Analog Device for Surgical Training and skills Reinforcement in Trichiasis
PGY: Postgraduate year
