# Supplementary material for: Evaluation and implementation of a mannequin-based surgical simulator for margin-involving eyelid laceration repair – a pilot study
Source: BMC Med Educ. 2021 Mar 19;21:170. doi: 10.1186/s12909-021-02600-3 (PMC7977496; doi:10.1186/s12909-021-02600-3)

**Evaluation and Implementation of a Mannequin-Based Surgical Simulator for Margin-Involving Eyelid Laceration Repair – A Pilot Study**

Jiawei Zhao, MD^a^; Meleha Ahmad, MD^a^; Emily W. Gower, PhD^b^; Roxana Fu, MD^c^; Fasika A. Woreta, MD, MPH^a^; Shannath L. Merbs, MD, PhD^d^

1. Wilmer Eye Institute, Johns Hopkins University School of Medicine, Baltimore, MD, USA
2. Gillings School of Global Public Health and Department of Ophthalmology, University of North Carolina, Chapel Hill NC, USA
3. Department of Ophthalmology, University of Pittsburgh Medical Center, Pittsburgh PA, USA
4. Department of Ophthalmology and Visual Sciences, University of Maryland School of Medicine, Baltimore MD, USA

**Corresponding Author:**

Shannath Merbs, M.D., Ph.D.

Department of Ophthalmology and Visual Sciences

University of Maryland School of Medicine

419 W. Redwood St., Suite 420

Baltimore, MD, 21201 USA

Telephone number: 667-214-1289

Fax number: 410-328-1178

Email: smerbs@som.umaryland.edu


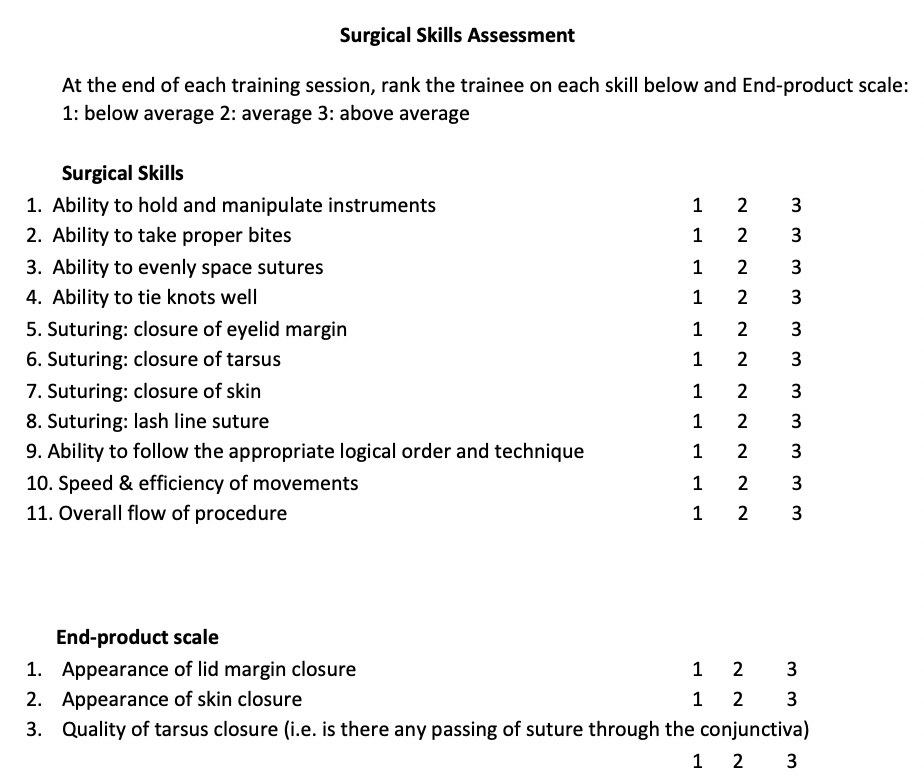

Supplement: Supplementary file 2 — Additional file 2: Supplementary file 2A. Surgical skills assessment tool for evaluating proficiency in performing margin-involving eyelid lacerations [file 12909_2021_2600_MOESM2_ESM.docx]
